# Virulence behavior of uropathogenic *Escherichia coli* strains in the host model *Caenorhabditis elegans*

**DOI:** 10.1002/mbo3.756

**Published:** 2018-10-31

**Authors:** Emily Schifano, Massimiliano Marazzato, Maria Grazia Ammendolia, Elena Zanni, Marta Ricci, Antonella Comanducci, Paola Goldoni, Maria Pia Conte, Daniela Uccelletti, Catia Longhi

**Affiliations:** ^1^ Department of Biology and Biotechnology Sapienza University Rome Italy; ^2^ Department of Public Health and Infectious Diseases, Microbiology Section Sapienza University Rome Italy; ^3^ National Center of Innovative Technologies in Public Health National Institute of Health Rome Italy

**Keywords:** *Caenorhabditis elegans*, *Escherichia coli*, oxidative stress, urinary tract infections, uropathogenic strains

## Abstract

Urinary tract infections (UTIs) are among the most common bacterial infections in humans. Although a number of bacteria can cause UTIs, most cases are due to infection by uropathogenic *Escherichia coli* (UPEC). UPEC are a genetically heterogeneous group that exhibit several virulence factors associated with colonization and persistence of bacteria in the urinary tract. *Caenorhabditis elegans* is a tiny, free‐living nematode found worldwide. Because many biological pathways are conserved in *C. elegans* and humans, the nematode has been increasingly used as a model organism to study virulence mechanisms of microbial infections and innate immunity. The virulence of UPEC strains, characterized for antimicrobial resistance, pathogenicity‐related genes associated with virulence and phylogenetic group belonging was evaluated by measuring the survival of *C. elegans* exposed to pure cultures of these strains. Our results showed that urinary strains can kill the nematode and that the clinical isolate ECP110 was able to efficiently colonize the gut and to inhibit the host oxidative response to infection. Our data support that *C. elegans*, a free‐living nematode found worldwide, could serve as an in vivo model to distinguish, among uropathogenic *E. coli*, different virulence behavior.

## 1 INTRODUCTION

Although *Escherichia coli* is an environmental colonizer and the predominant nonpathogenic facultative anaerobic constituent of the intestinal microbiota of warm blooded animals, some strains are able to cause diseases in humans as well as in mammals and birds (Dho‐Moulin & Fairbrother, 1999; Kaper, Nataro, & Mobley, 2004). *E. coli* pathotypes found in humans can be categorized into diarrheagenic and extraintestinal pathogenic *E. coli* (ExPEC) (Croxen et al., 2013; Kӧhler & Dobrindt, 2011). ExPEC, a heterogeneous group causing a diversity of infections outside the intestinal tract in several animal hosts, includes neonatal meningitis *E. coli* K1 (NMEC) and human uropathogenic *E. coli* (UPEC) (Ewers et al., 2007; Johnson et al., 2008). UPEC is the primary cause of community and nosocomial‐acquired urinary tract infections (UTIs), accounting for substantial medical costs and morbidity worldwide. UPEC is associated with acute as well as chronic and recurrent infections that require long‐term antibiotic therapy and are often associated with life‐threatening sequelae (Blango & Mulvey, 2010; Foxman, 2003; Soto et al., 2007).

Uropathogenic *E. coli* produces numerous virulence factors, including various adhesins, iron chelators, capsule‐forming polysaccharides, flagella, and toxins (e.g., hemolysin, cytotoxic necrotizing factor 1), which enable UPEC to colonize and manipulate the host innate immune response (Johnson, 1991; Johnson & Russo, 2002). The ability of UPEC to invade and multiply in host epithelial cells and form biofilms also enhances UPEC virulence and persistence within the urinary tract (Chen, Xiong, Sun, Yang, & Jin, 2012).

In *E. coli* causing UTIs, no distinctive virulence factor separates UPEC from non‐UPEC strains. Furthermore, a distinction between ExPEC and commensals is not straightforward, as strains with the ability to cause extraintestinal infections belong to the normal enteric flora of many healthy individuals (Barnich & Darfeuille‐Michaud, 2007; Martinez‐Medina et al., 2009). To assume that an *E. coli* isolate is ExPEC many features should be considered: clinical context and source of isolation, characterization of the isolate for phylogenetic background as well as testing the isolate in an animal infection model (Hagberg et al., 1983; Johnson et al, 2008). Phylogenetic analysis has shown that *E. coli* strains fall into four main groups (A, B1, B2, and D). It has been found that pathogenic *E. coli* strains causing extraintestinal infections mainly belong to group B2 and a lesser extent to group D whereas commensal strains belong to group A and B1 (Dale & Woodford, 2015).

Furthermore, surface water, rainwater, sewage, wastewater effluents, wild animals, and soil have all been investigated as possible environmental sources of ExPEC, and different studies have tried to associate virulence and antibiotic resistance traits to environmental *E. coli* clones (Amos, Hawkey, Gaze, & Wellington, 2014; Anastasi et al., 2010; Manges & Johnson, 2015; Müller, Stephan, & Nüesch‐Inderbinen, 2016).

Among in vivo models, *Caenorhabditis elegans* have been proposed as a model to study phenotypic and genotypic virulence determinants of ExPEC (Diard et al., 2007). *C. elegans*, an ubiquitous free‐living nematode which lives in soil and feeds on bacteria, sharing with humans many biological pathways, has become a widely used model organism for studying host interactions with microbes and virulence mechanisms of microbial infections (Anyanful et al., 2005; Barber, Norton, Wiles, & Mulvey, 2016; Burton, Pendergast, & Aballay, 2006; Mylonakis, Ausubel, Tang, & Calderwood, 2003). It has been reported that free‐living nematodes may serve as carriers or vectors of human enteric pathogens from soil resources, and these nematodes have been shown to be resistant to free chlorine and to offer protection to ingested pathogens against chemical sanitizers (Caldwell, Adler, Anderson, Williams, & Beuchat, 2003; Merkx‐Jacques et al., 2013).

In this study, in vitro and in vivo approaches were utilized to evaluate the behavior of two clinical *E. coli* UPEC isolates.

## 2 MATERIALS AND METHODS

### 2.1 *Escherichia coli* strains

The studied *E. coli* derived from a collection of UPEC strains isolated from urine of inpatients in a tertiary teaching hospital in Rome. Bacterial identification to the species level was performed by an automated Vitek 2 instrument (bioMérieux).

ECP45 was isolated from a patient in a medical ward, with uncomplicated UTI, and ECP110 derived from a catheterized patient (CAUTI) in the neurological intensive care unit.

*Escherichia coli* strains, isolated on McConkey agar (Oxoid, Rome, Italy), were grown in Luria broth (LB) or Mueller‐Hinton broth (MHB) (Oxoid) and stored in glycerol at −80°C. The *E. coli* K12 MG1655 (Guyer, Reed, Steitz, & Low, 1981) and the uropathogenic *E. coli* CFT073 strain, isolated from blood of a patient suffering from acute pyelonephritis, were used as controls (Mobley et al., 1990).

### 2.2 Antimicrobial susceptibility testing

The antibiotic susceptibility test was performed by Vitek 2 System (BioMèrieux). Results were interpreted by the Advanced Expert System software (AST‐N202) using current EUCAST break point (2015 Clinical break points—bacteria v 5.0, www.eucast.org/ast).

### 2.3 Phylogenetic grouping, multilocus sequence type analysis, and virulence genotyping

For each *E. coli* strain, phylogenetic grouping (A, B1, B2, and D) was determined by a triplex PCR, which uses a combination of three DNA markers (chuA, yjaA, and the DNA fragment TspE4.C2) as developed by Clermont, Bonacorsi and Bingen (2000). All PCRs were performed in duplicate with appropriate positive and negative controls. Multilocus sequence type (MLST) analysis was performed by amplifying fragments of seven housekeeping genes as previously described (Wirth et al., 2006). The sequences relative to fragments were obtained by standard sequencing techniques and, subsequently, the sequence type (ST) of each strain was determined by comparison with the specific database hosted at https://enterobase.warwick.ac.uk/species/ecoli/allele_st_search. Phylogenetic relationships of the two isolates and *E. coli* strains from different origin, whose genome was publicly available, were evaluated. For this purpose genomes relative to the intestinal origin *E. coli* strains MG1655 (NC_000913.3), DH10b (NC_010473.1), ATCC8739 (NC_010468.1), HS (NC_009800.1), to the UPEC strains CFT073 (NC_004431.1), UTI89 (NC_007946.1), 536 (NC_008253.1), JJ1886 (NC_022648.1) and to the environmental origin strain SMS‐3‐5 (NC_010498) were downloaded as fasta files from GenBank and imported in Geneious R 7.1.3 (Biomatters, New Zealand). An in silico PCR for MLST specific genes was performed by using previously reported primer sequences (Wirth et al., 2006). For each strain, the MLST genes were concatenated and aligned by using MUSCLE (Edgar, 2004). A minimum spanning three (MST) was constructed by using Phylowiz with goeBURST algorithm on concatenated allele sequences (Francisco, Bugalho, Ramirez, & Carrico, 2009).

Multiplex PCR for selected pathogenicity‐related genes associated with virulence in *E. coli*, was performed (Johnson & Stell, 2000).

### 2.4 Oxidative stress resistance

A disk diffusion assay was performed to determine the sensitivity of various *E. coli* strains to the reactive oxygen species (ROS), hydrogen peroxide. Overnight LB bacterial cultures were suspended in PBS to OD_600_ of 0.5 and spread (about 10^5^ CFU, Colony Forming Units) over LB agar plate. Filter paper disks (6 mm; Becton Dickinson) were placed on the surface, and 10 μl of hydrogen peroxide (30% [vol/vol] or diluted) was loaded onto each disk. After overnight growth at 37°C, the diameters of inhibition zones were measured.

### 2.5 Blood agar plate assay

Bacterial strains were streaked onto blood agar plates containing 5% defibrinated sheep blood. The plates were examined up to 48 hr of incubation at 37°C for the presence of hemolysis area around colonies (Beutin et al., 1989).

### 2.6 Microtiter plate biofilm production assay

Cultures (20 µl 1–2 × 10^8^ CFU/ml) were inoculated into wells of a 96‐well polystyrene plate containing 180 μl of LB. After 48 hr at 26°C, the wells were rinsed with phosphate buffered saline and allowed to dry. Bacterial cells bound to the wells were stained with crystal violet (Sigma‐Aldrich, 1% w/v) for 15 min. The dye bound to the adherent bacterial cells was solubilized with 95% (v/v) ethanol for 15 min. *E. coli* ATCC 25922 and the UPEC strain 16 (Longhi et al., 2016) were used as biofilm positive and negative controls, respectively. The optical density (OD) at 570 nm of each well was measured, and biofilm production was classified as described by Stepanović, Cirković, Ranin, and Svabić‐Vlahović (2004). Based on the OD produced by bacterial films, strains were classified into the following categories: non‐producers, weak, moderate, or strong biofilm producers. The cut‐off OD was defined as three standard deviations above the mean OD of the negative control (ODc). Biofilm production was classified as follows: OD ≤ ODc = no biofilm formation, ODc < OD ≤ (2 × ODc) = weak biofilm formation, (2 × ODc) < OD ≤ (4 × ODc) = moderate biofilm formation, and (4 × ODc) < OD = strong biofilm formation.

### 2.7 Cell line

The HEp‐2 human epithelial cell line (ATCC CCL23, American Type Culture Collection, MD, USA) was routinely maintained in Eagle's minimal essential medium (MEM; Sigma Chemical Co., USA), supplemented with 10% fetal bovine serum (FCS; Gibco‐BRL), penicillin (10 U/ml), and streptomycin (10 μg/ml). Cells were maintained at 37°C in 5% CO_2_.

### 2.8 Bacterial adhesion to and invasion of HEp‐2 cell line

Adhesion and invasion assays were performed with slight modifications as previously described (Longhi et al., 2016). Briefly, HEp‐2 cell monolayers, cultured in 24‐well plate at a density of 2 × 10^5^ cells/well for 24 hr at 37°C in 5% CO_2_, were infected by adding logarithmically grown *E. coli* strains at a multiplicity of infection of approximately 10 bacteria per cell (MOI: 10) then centrifuged twice at 500 *g* for 2.5 min to synchronize infection and incubated for 1 hr at 37°C in 5% CO_2_ (Thumbikat et al., 2009). After five washes in MEM to remove unattached bacteria, cells were lysed adding ice‐cold 0.1% Triton X‐100. Bacteria were counted on Tryptone Soya Agar plates. Bacterial adhesion was defined as the percentage of attached bacteria compared with the initial inoculum. The invasive ability was measured using the gentamicin protection assay. After the infection period, monolayers were extensively washed with PBS, incubated for an additional hour in culture medium supplemented with 100 µg/ml gentamicin to kill extracellular bacteria, then lysed as above. In survival and multiplication assays, after the incubation time, medium containing 50 µg/ml gentamicin was added, and multiplication was evaluated at 3 and 24 hr postinfection. Cells were then lysed, and bacteria were plated onto agar. All assays were performed in triplicate. *E. coli* MG1655 was used as negative control.

### 2.9 Nematode strains and maintenance

The *C. elegans* strains used in this study are the Bristol N2 as standard wild type strain and the CF1553 (*muls84[pAD76(Sod‐3::GFP)]*) transgenic strain, from Caenorhabditis Genetic Center. Strains were grown at 16°C on Nematode growth medium (NGM) plates with fresh *E. coli* OP50 as standard laboratory food (Brenner, 1974).

### 2.10 *Caenorhabditis elegans* infections

*E. coli* cultures were grown exponentially in LB at 37°C. Bacterial lawns used for *C. elegans* infection assays were prepared by spreading 30 μl of each culture corresponding to 1 × 10^8^ cells on the NGM agar plates (35 mm). The plates were incubated at 37°C for 24 hr before being seeded with young adult nematodes, grown at 16°C, from a synchronized culture (Brenner, 1974). The infections were performed at 25°C for several days, as indicated. Worms were transferred daily on new freshly‐prepared plates. Worm death was scored by the absence of touch‐provoked movement. Heat‐killed bacterial cells were prepared as follows. Overnight cultures, prepared as above, were incubated at 65°C for 90 min and deposited onto NGM agar plates. Heat‐killed cells were also plated on LB agar in parallel to ensure that no viable cells remained.

### 2.11 Estimation of bacterial CFU within the nematode gut

The numbers of live *E. coli* bacteria in the worm intestine were determined after 48 hr of infection, as described (Zanni et al., 2015). Briefly, for each sample, 10 infected worms were washed three times with 500 μl of M9 buffer to remove bacteria on nematode surface. Worms were broken with 100 μl of M9 buffer‐1% Triton X‐100; lysates were diluted and then plated on LB agar plates. The CFU were counted after overnight incubation of LB agar plates at 37°C.

### 2.12 Fluorescence microscopy analysis of nematodes

For the colonization analysis, N2 worms were infected for 48 hr with the *E. coli* strains transformed with the GFP‐expressing plasmid pFPV25.1 (Valdivia & Falkow, 1996). Then, nematodes were mounted onto 3% agarose pads containing 20 mM sodium azide and observed with a Zeiss Axiovert 25 microscope with a standard filter set. For the oxidative stress experiments, the transgenic SOD3::GFP strain was infected with the bacterial strains for 48 hr and then observed under the above‐mentioned microscope. Quantification of fluorescence intensity was evaluated with ImageJ 1.43 (NIH) software measuring the ratio of pixels per area of worm. For each sample, 10 transgenic nematodes were analyzed and the mean value was reported.

### 2.13 Oxidative stress analysis in *C. elegans*

Reactive oxygen species formation in *C. elegans* was measured using the fluorescent probe H_2_DCFDA according to Kampkötter et al. (2007) with minor modifications. Briefly, N2 synchronized adult worms were exposed to *E. coli* strains for 48 hr. Worms were then collected (in triplicate) into 0.5 ml of M9, washed three times and transferred into wells of a 96‐well microtiter plate containing 0.1 ml of 50 μM H_2_DCFDA. Fluorescence was read immediately and 120 min after addition, by using a microplate reader at excitation/emission wavelengths of 485 and 520 nm. Initial readings were subtracted from the final readings, and mean fluorescence was calculated from the triplicate. Results are expressed as ROS levels relative to *E. coli* CFT073‐fed worm (control) and are the mean of three independent experiments.

### 2.14 Statistical analysis

Data are presented as mean ± SD, one‐way ANOVA analysis followed by post hoc *T* test with Bonferroni's correction for multiple comparisons (GraphPad Prism 5.0 software) was used to determine the statistical significance between experimental groups. Statistical significance was defined as *p < 0.05, **p < 0.01, and ***p < 0.001.

## 3 RESULTS

### 3.1 In vitro assays

The preliminary screening based on antibiotic resistant pattern and phylogenetic groups was performed on UPEC strains isolated from urine of inpatients in a tertiary teaching hospital in Rome. Antimicrobial assay showed that ECP110 strain possessed a fully susceptible profile to fluoroquinolones, aminoglycosides, trimethoprim‐sulfamethoxazole, penicillins, and tetracycline; ECP45 strain was resistant only to tetracycline (Table 1); the different kind of patient/infection (uncomplicated UTI vs. CAUTI) and the lack of resistance to most antibiotic classes, associated more often to environmental strains, prompted us to choose ECP45 and ECP110 strains to be further characterized. Regarding the phylogenetic group, ECP45 and ECP110 belonged to group D and B2 respectively while MLST analysis showed that ECP45 belonged to ST362 and ECP110 to ST357. Out of virulence factors tested, the difference regarded capsular genes. Only ECP110 expressed lysis ability in blood agar plates although delayed at 48 hr, even though both strains were positive for *hly* gene (Table 1).

**Table 1:** Characteristics of UPEC strains

	Strains		
	ECP110	ECP45	CFT073
Clinical source	Urinary tract infection	Urinary tract infection	Blood pyelonephritic
Antibiotic resistance[Link]	Fully susceptible	Tetracyclin	Aminoglycosides
Phylogenetic group	B2	D	B2
Virulence genes			
*papACEFG*	+	+	+
*sfa/focDE*	−	−	+
*fimH*	+	+	+
*hlyA*	+	+	+
*cnf1*	−	−	−
*fyuA*	+	+	+
*iutA*	−	−	+
*ibeA*	−	−	−
*traT*	+	+	−
*kpsMTII*	+	−	+
*kpsMTIII*	−	+	−
Multilocus sequence type	357	362	73

Note

UPEC: uropathogenic *Escherichia coli*.

Antibiotic class considered: fluoroquinolones, trimethoprim‐sulfamethoxazole, penicillins, tetracycline, and aminoglycosides.

**Figure 1:**
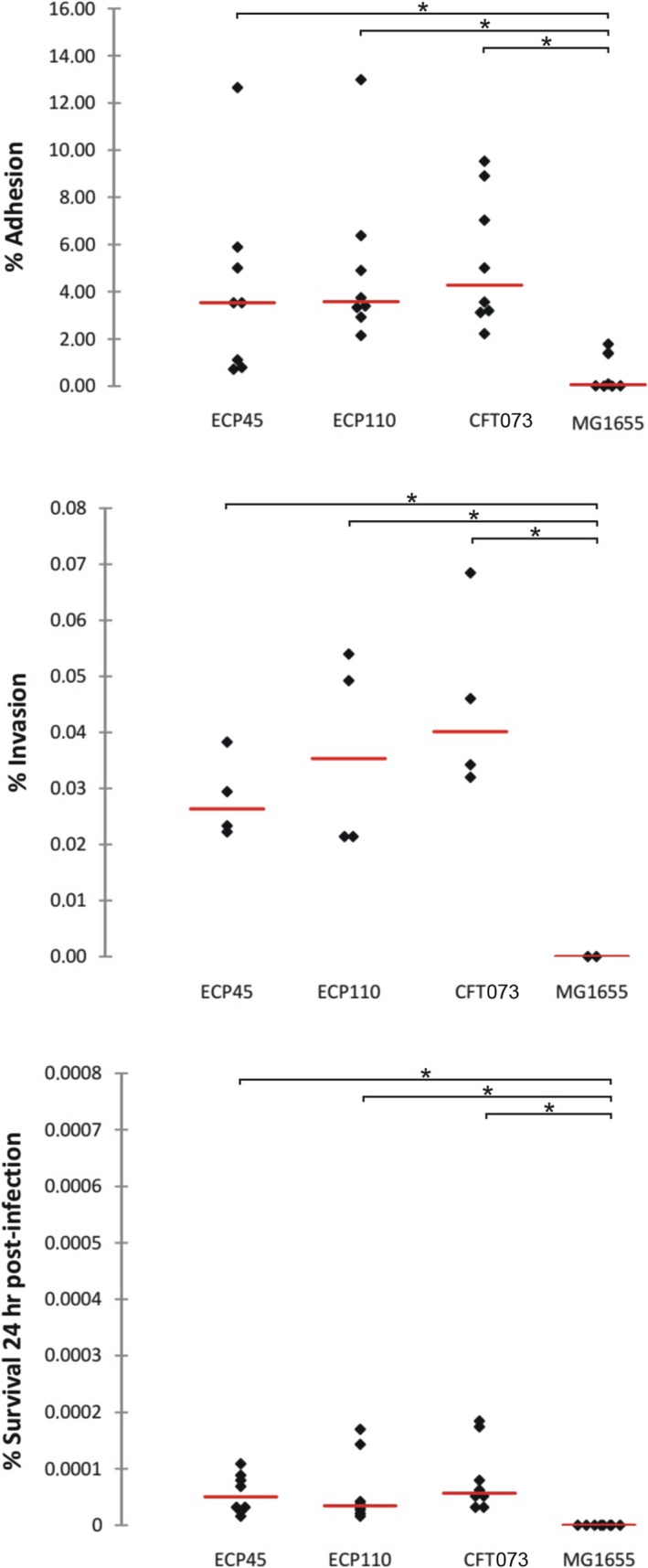
HEp‐2 cell monolayers were infected by adding logarithmically grown of *Escherichia coli* strains at a multiplicity of infection of approximately 10 bacteria per cell. Bacterial invasion was measured using a gentamicin protection assay before lysis and plating as described in Materials and methods (*p < 0.05)

Biofilm formation assay showed that all strains were able to produce biofilm on abiotic surfaces at different extent. *E. coli* CFT073 strain was the most efficient biofilm producer (OD 1.0 ± 0.36); ECP45 and ECP110 were moderate and weak producer (OD 0.37 ± 0.09 and OD 0.30 ± 0.11, respectively). The relative sensitivity of *E. coli* strains to ROS was also evaluated. The strain MG1655 was more susceptible to the ROS‐generating compound hydrogen peroxide than the UPEC strains as determined by a disk diffusion assay (data not shown). UPEC strains were able to adhere to and invade HEp‐2 cells to a similar extent; after 24 hr from infection, the number of internalized UPEC bacteria appeared drastically reduced (Figure 1).

**Figure 2:**
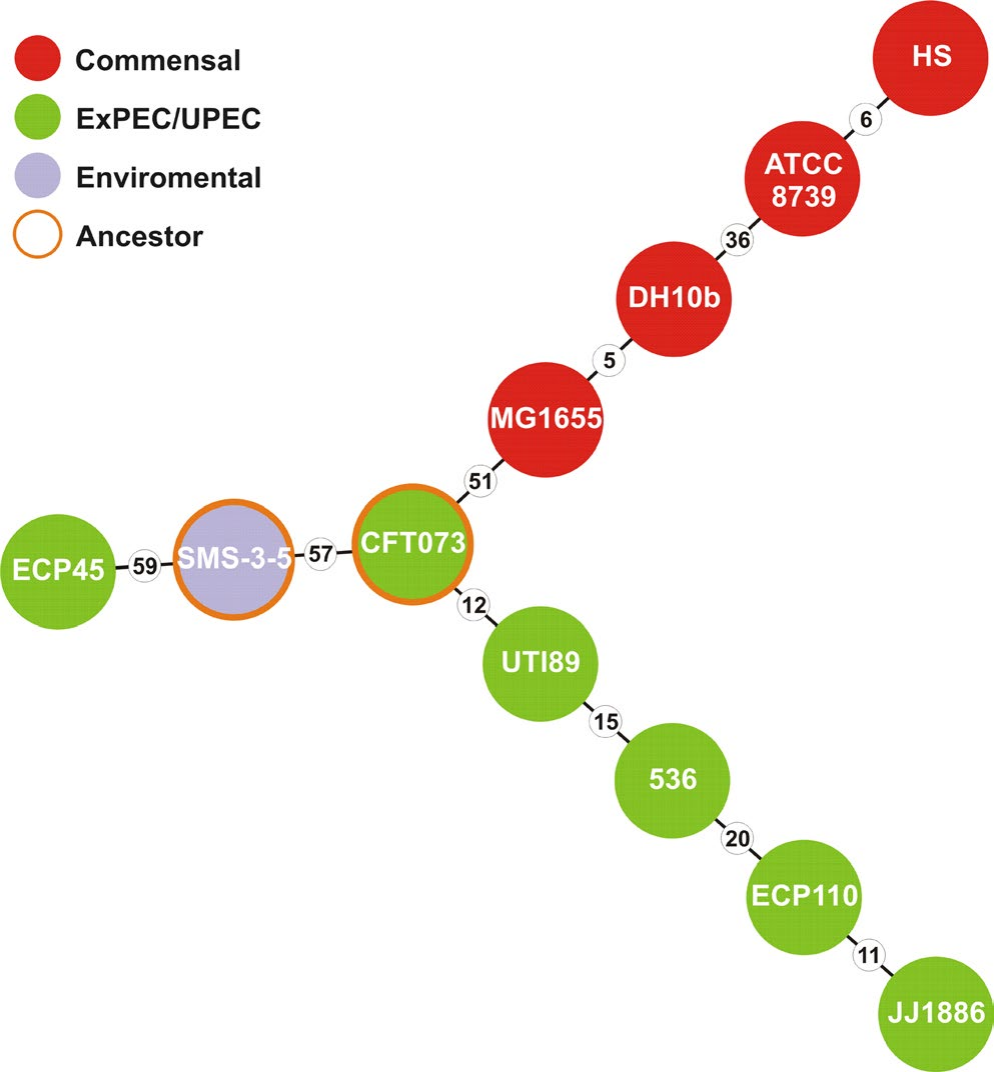
Minimum spanning tree based on multilocus concatenated alleles showing the phylogenetic relationship between ECP45 and ECP110 strains and uropathogenic *Escherichia coli* (UPEC), commensal and environmental origin *E. coli* strains. Genetic distances, expressed as amount of single nucleotide variants, are showed as number in small circles

Results relative to in vitro MLST analysis of both ECP45 and ECP110 strains as well as in silico MLST assays of well known UPEC, commensal and environmental origin strains were showed (Figure 2).

The MST constructed on concatenated alleles indicates that ECP110 was phylogenetically related to different UPEC reference strains while ECP45 to the environmental *E. coli* strain SMS‐3‐5 (Fricke et al., 2008).

### 3.2 In vivo assays

**Figure 3:**
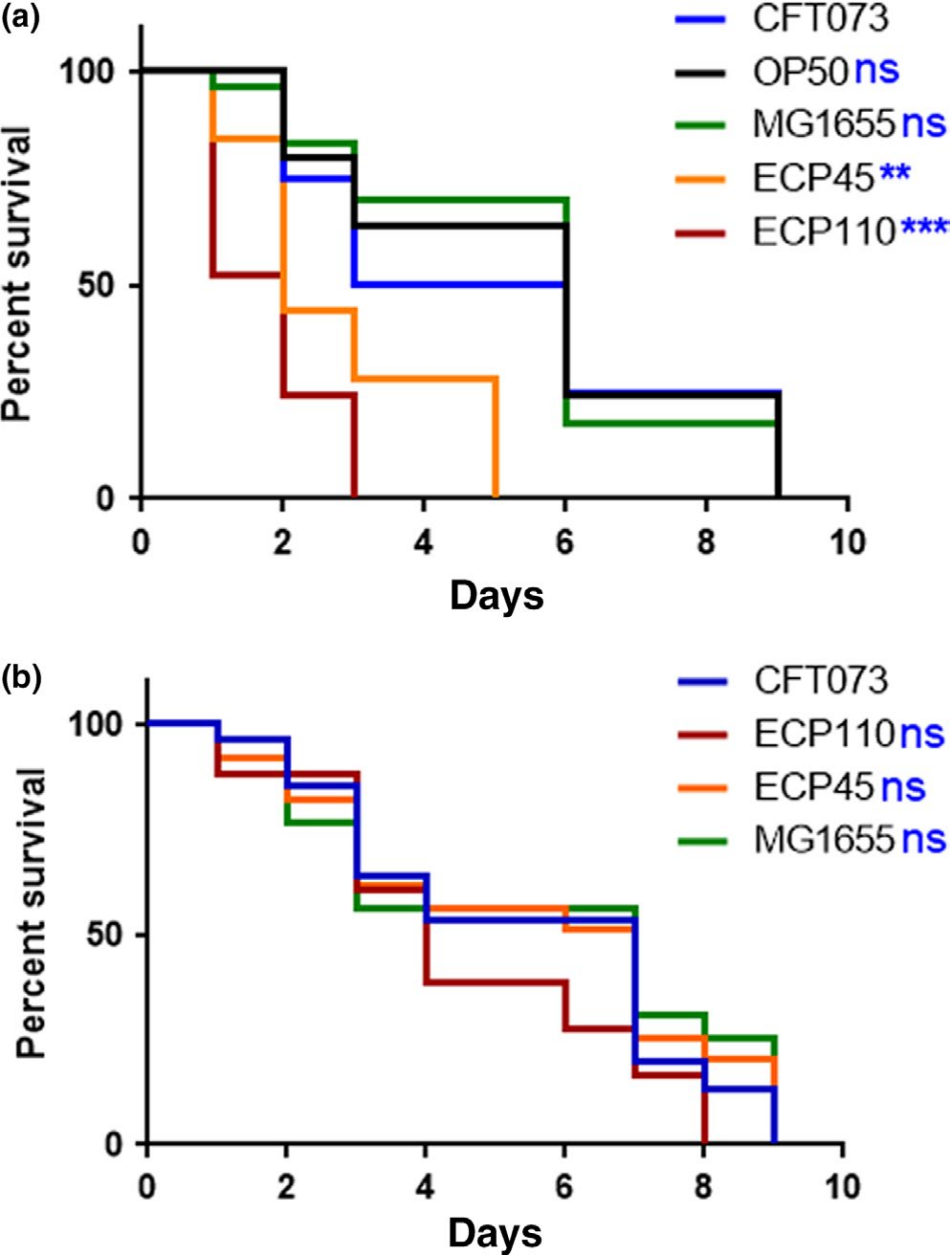
(a) Kaplan–Meier survival plots of worms infected with the indicated *Escherichia coli* strains are shown. Infections were performed at 25°C, and worm mortality was monitored every day. *E. coli* CFT073‐fed worms were taken as control. (b) Survival of *Caenorhabditis elegans* fed heat‐killed *E. coli* strains. *n* = 60. Statistical analysis was evaluated by Log‐rank (Mantel‐Cox) test; asterisks indicate significant differences (**p < 0.01; ***p < 0.001; ns: not significant)

The *C. elegans* model was exploited to compare the in vivo virulence of the UPEC isolates, ECP45 and ECP110. To this aim, an infection assay was performed by measuring the survival of *C. elegans* fed on pure cultures of these strains; *E. coli* strains OP50 (standard *C. elegans* laboratory food) and CFT073 were used as controls. Infection data (Figure 3a) showed that after 24 hr almost 50% of ECP110‐fed worms were dead evidencing that ECP110 strain was significantly more virulent than both ECP45 and the control strain (survival >50% at 6 days). Notably, the life span shortening was dependent on the viability of the urinary strains used as food source. Indeed, dietary administration of heat‐killed UPEC did not produce any effect on nematode lifetime (Figure 3b) indicating that viability of the strain was required for pathogenicity.

**Figure 4:**
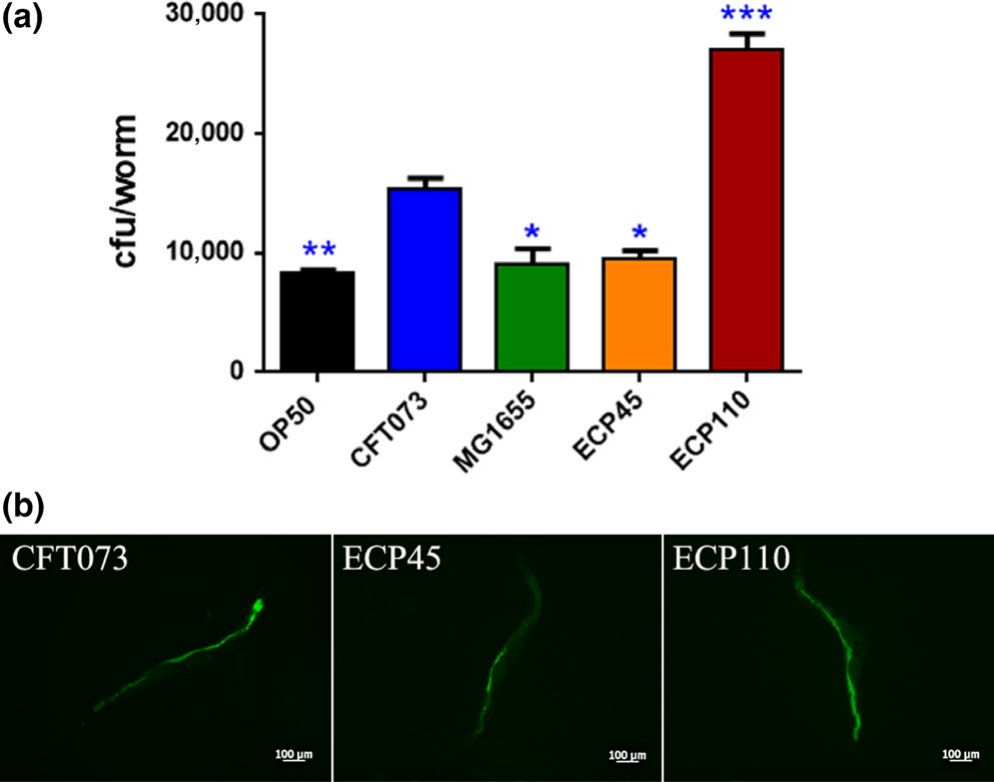
(a) Colonization of uropathogenic *Escherichia coli* strains within the nematode gut. coli strains within the nematode gut. Asterisks indicate significant differences (^*^ *p* < 0.05; ^**^ *p* < 0.01; ^***^ *p* < 0.001). (b) Fluorescence photomicrographs of 10 representative nematodes infected with the GFP‐expressing CFT073, ECP45 and ECP110 strains for 2 days are reported (scale bar, 100 µm)

Afterward, the quantity of intestinal bacteria within *C. elegans* gut was analyzed through CFU method after 2 days of infection. ECP110 strain showed a colonization capacity almost 2‐ and about 3‐fold higher than CFT073 and OP50 controls, respectively (Figure 4a). To visualize the bacterial colonization in the nematode intestinal tract, the GFP‐expressing *E. coli* strains were then used to feed *C. elegans*. After a 48 hr‐exposure to infection, a high fluorescence was observed in animals fed with ECP110–GFP strain, where bacterial cells had spread along the entire nematode gut (Figure 4b), highlighting a well evident difference when compared to ECP45‐GFP or CFT073‐GFP infections.

Different studies in *C. elegans* reported that exposure to pathogens, including *Pseudomonas aeruginosa* and *Enterococcus faecalis*, led to oxidative inducing tissue‐damage, especially in the intestine cells, caused by both bacterial virulence factors and host antimicrobial defense mechanisms (Chávez, Mohri‐Shiomi, Maadani, Vega, & Garsin, 2007; Mahajan‐Miklos, Tan, Rahme, & Ausubel, 1999).

**Figure 5:**
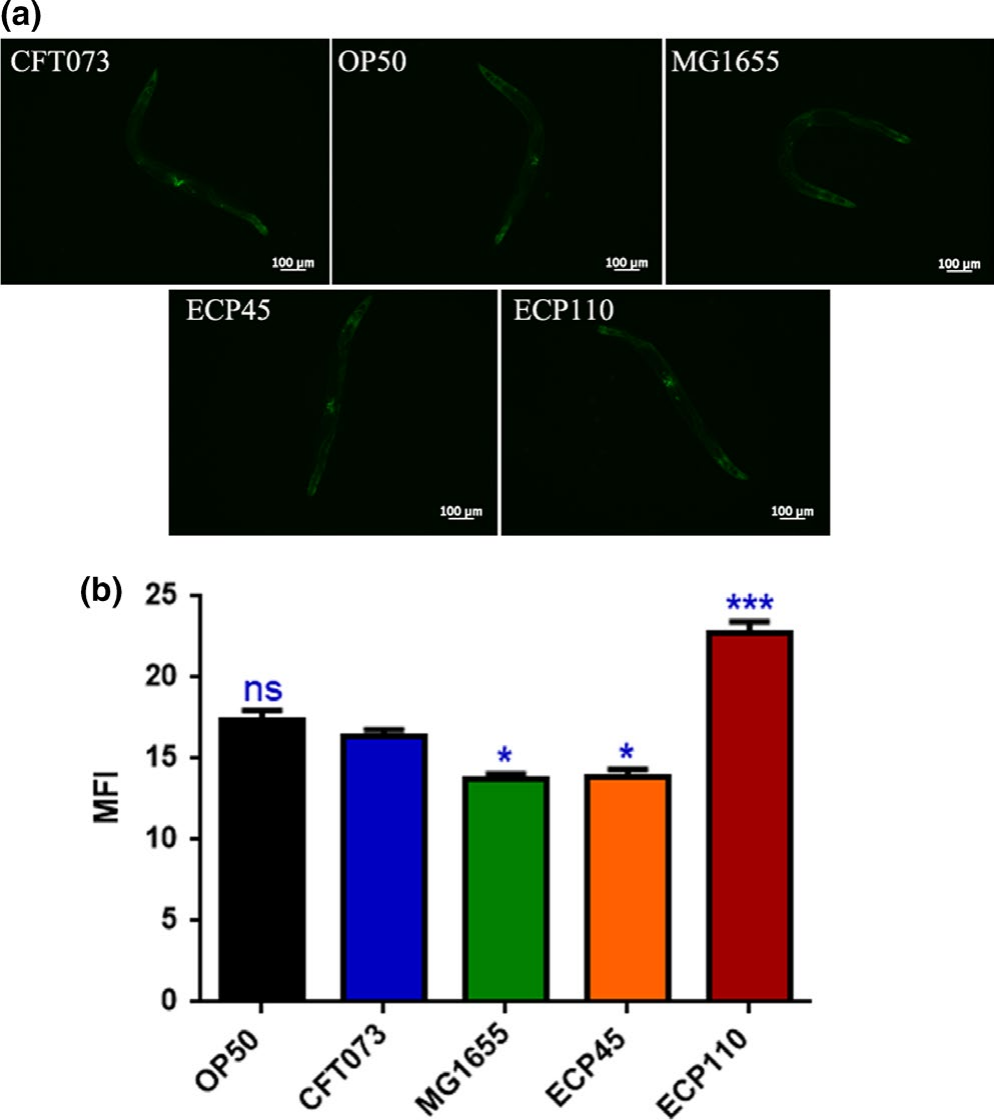
(a) Fluorescence microscopy of SOD3::GFP worm strain after 48 hr of *Escherichia coli* strains infection. Scale bar = 100 μm. (b) MFI represents mean fluorescence intensity of *Caenorhabditis elegans* SOD3::GFP transgenic strain fed different *E. coli* strains (*p < 0.05; ***p < 0.001; ns: not significant)

**Figure 6:**
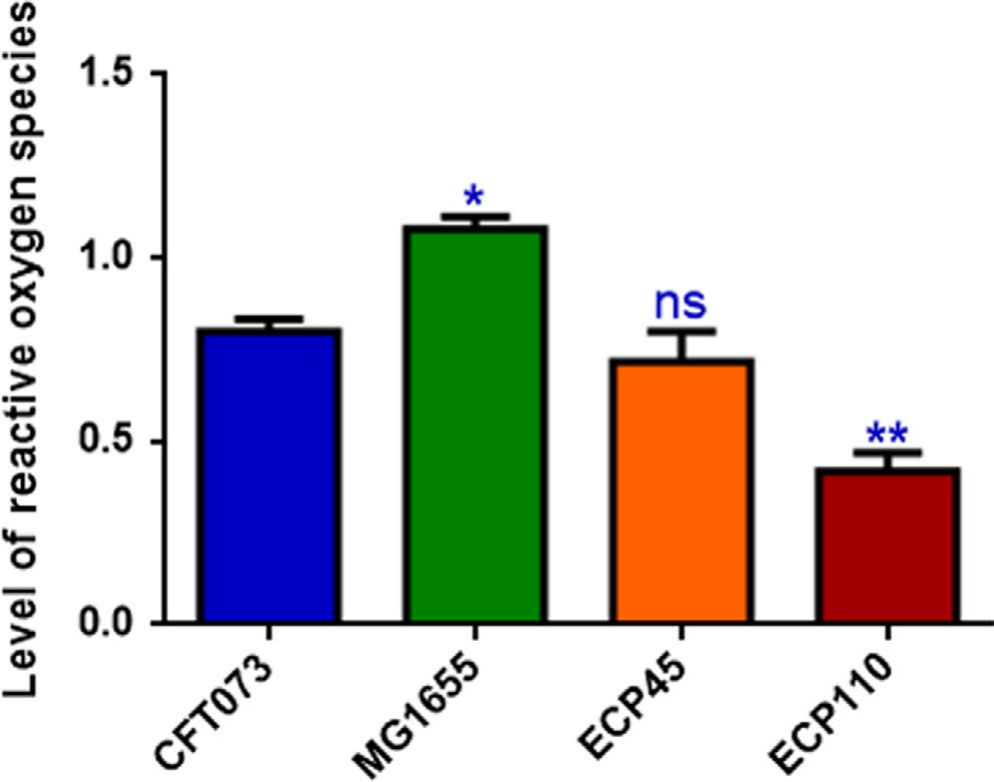
ROS production in H_2_DCFDA‐stained N2 worms after exposure to pathogenic *Escherichia coli* strains for 2 days. Statistical analysis was performed with respect to animals fed with CFT073 strain (*p < 0.05; **p < 0.01; ns: not significant)

To test whether oxidative stress response took place in UPEC‐infected worms, we made use of the transgenic *C. elegans* CF1553 strain, expressing GFP as a reporter transgene for an iron/manganese superoxide dismutase expression. Notably, transgenic animals exposed to ECP110 cells for 48 hr showed significant differences in fluorescence level respect to control strains, unlike ECP45 and MG1655 strains (Figure 5a,b). Based on that data, ROS production was then evaluated in nematodes infected with *E. coli* isolates. ROS levels relative to ECP110 diet were lower than those relative to other *E. coli* strains, suggesting that no ROS accumulation was observed after infection with that urinary strain (Figure 6).

## 4 DISCUSSION

*Caenorhabditis elegans* has been used as a model for infections caused by different human pathogens, since many of which give rise to infectious processes comparable, at the cellular level, to those developed in humans (Powell & Ausubel, 2008). Among them, effects exerted by the Gram‐negative *P. aeruginosa* and the Gram‐positive *Staphylococcus aureus* were well studied in nematodes (King et al., 2018; Pidgeon & Pires, 2017; Sifri, Begun, Ausubel, & Calderwood, 2003; Tan, Mahajan‐Miklos, & Ausubel, 1999).

In this study, virulence of *E. coli* isolates from urinary infections was evaluated in *C. elegans* model. The survival of worms fed on pure cultures of these strains was measured: uropathogenic ECP110 was more virulent with respect to ECP45 isolate and the prototypic UPEC CFT073 and MG1655 strains.

Diard et al. (2007) established a link between bacterial virulence in *C. elegans* and certain phenotypic and genetic predictors of ExPEC pathogenicity.

Concerning the presence of virulence factors, both bacterial strains possessed *hly*A gene, although ECP110 strain showed a delayed hemolytic activity. Different reports indicate that UPEC strains that express HlyA were associated with more severe clinical outcomes (Merkx‐Jacques et al., 2013). In the epithelial cell line HEp‐2, the in vitro model utilized in this study, both ECP45 and ECP110 were able to adhere and invade to a extent and they were unable to survive in cell monolayers. Adherence capacity of *E. coli* to HEp‐2 cells was unlikely to be associated with mice uroepithelial adherence as reported by Kim and Lee (2017). Likewise in our study, UPEC strains adhere to HEp‐2 cell monolayers in a similar extent but only ECP110 resulted able to colonize the worm gut. Moreover, Brzuszkiewicz et al. (2006) reported that individual differences among UPEC, including CFT073, in their potential to cause disease and in the severity of the UTI, seem to be the result of presence/expression of common as well as strain‐specific gene sets. Overall these data highlight the need to use several models to investigate the main aspects of host‐pathogen interactions.

As reported by different Authors UPEC strains have a clear competitive advantage during biofilm growth on catheter surfaces (Ferrières, Hancock, & Klemm, 2007). CAUTI isolate ECP110 showed a weak biofilm production ability in comparison with CFT073, a good producer strain on abiotic surfaces. However, ECP110 was able to more efficiently colonize the gut than ECP45 and CFT073 strains, suggesting that different factors were involved.

Possibly, ECP110 efficiency to colonize worm gut could be due to the bacterial ability to cause the decrease of ROS levels in *C. elegans* showing possible pathobiont behavior. Pathobionts are members of the microbiota that have the ability to promote immune maturation or inflammation (Hornef, 2015). Some of these have different capacity to modulate ROS, and they could have functional consequences in the host organism. For example, it has been demonstrated that the expansion of a commensal *E. coli* strain, resistant to ROS, predisposed mice to infection by *Vibrio cholerae* (Mi et al., 2016).

In response to the breach by UPEC into urinary tract, a strong host innate response is triggered. The host inflammatory response leads to the exfoliation of infected bladder epithelial cells and generation of reactive nitrogen and oxygen species with other antimicrobial compounds. The ability of some UPEC strains to delay or to suppress innate immune response could play a role in persistence of pathogen within urinary tract (Mulvey, Schilling, Martinez, & Hultgren, 2000). UPEC ECP110 evasion system may constitute an critical event in colonization of the urinary tract.

Various signaling pathways activate ROS and antimicrobial mechanisms, enhancing the expression of proteins involved with these pathways. Since the production of ROS is a microbicidal mechanism, many pathogens downregulate the expression or interfere with the activity of downstream effectors (Paiva & Bozza, 2014). It has been reported that *C. elegans* intestinal infection triggers the release of ROS, as a general protective response that most animals possess (Chávez et al., 2007; Chávez, Mohri‐Shiomi, & Garsin, 2009).

Normally, in order to avoid the harmful effects of ROS, *C. elegans* activated pathogen‐specific host response through induction of genes encoding ROS detoxifying enzymes, such as superoxide dismutases (Hoeven, McCallum, Cruz, & Garsin, 2011). Indeed, it has been reported that the superoxide dismutase SOD‐3 protects worms from oxidative stress (Doonan et al., 2008).

However, we found a reduction of ROS levels when nematodes were infected with ECP 110 with respect to the CFT073 strain, correlating with a higher expression levels of the oxidative stress response gene *sod‐3.* We may thus speculate that the modulation of the worm oxidative response may in part account for the higher virulence exerted by ECP110 strain.

Nematodes are normally found in compost‐rich soil and migrate throughout the soil matrix, searching for bacteria as the main food source. A fraction of the ingested bacteria can reach the worm bowel as living microorganisms that can colonize the gut. As already suggested (Brennan, Abram, Chinalia, Richards, & O'Flaherty, 2010), all together our data indicate that the high colonization capability of ECP110 of *C. elegans* gut, followed by death of the worms, could permit bacterial survival, protection by stress so that promoting environmental spread.

## CONFLICT OF INTEREST

The authors declare no conflict of interest.

## AUTHORS CONTRIBUTION

CL, DU, AC, and MGA conceived and designed the experiments. DU, CL, and AC wrote the paper. ES and EZ did nematode experiments. MM and MR did microbial experiments. PG and MPC involved in critical revision of manuscript.

## ETHICS STATEMENT

Not required.

## Data Availability

All data are included in the main manuscript. Raw data and materials are available on request.
